# High-Resolution Profiling of Gut Bacterial Communities in an Invasive Beetle using PacBio SMRT Sequencing System

**DOI:** 10.3390/insects10080248

**Published:** 2019-08-14

**Authors:** Letian Xu, Liuwei Sun, Shihan Zhang, Shanshan Wang, Min Lu

**Affiliations:** 1 State Key Laboratory of Biocatalysis and Enzyme Engineering, School of Life Sciences, Hubei University, Wuhan 430062, China; 2 Cardiff Sixth Form College, Cardiff CF24 0AA, UK; 3 Institute of Physical Science and Information Technology, Anhui University, Hefei 230601, China; 4 State Key Laboratory for Biology of Plant Diseases and Insect Pests, Institute of Plant Protection, Chinese Academy of Agricultural Sciences, Beijing 100193, China

**Keywords:** *Dendroctonus valens*, bark beetle, gut bacteria, PacBio, high-resolution profiling, ontogeny

## Abstract

*Dendroctonus valens*, an invasive bark beetle, has caused severe damage to Chinese forests. Previous studies have highlighted the importance of the gut microbiota and its fundamental role in host fitness. Culture-dependent and culture-independent methods have been applied in analyzing beetles’ gut microbiota. The former method cannot present a whole picture of the community, and the latter mostly generates short read lengths that cannot be assigned to species. Here, the PacBio sequencing system was utilized to capture full-length 16S rRNA sequences in *D. valens* gut throughout its ontogeny. A total of eight phyla, 55 families, 102 genera, and 253 species were identified. Bacterial communities in colonized beetles have the greatest richness but the lowest evenness in all life stages, which is different from those in young larvae. *Pseudomonas* sp., *Serratia liquefaciens* possess high abundance throughout its ontogeny and may serve as members of the core bacteriome. A phylogenetic investigation of communities by reconstruction of unobserved states (PICRUSt) analysis predicted that gut microbiota in larvae are rich in genes involved in carbohydrate, energy metabolism. Gut microbiota in both larvae and colonized beetles are rich in xenobiotics and terpenoids biodegradation, which are decreased in dispersal beetles. Considering that the results are based mainly on the analysis of 16S rRNA sequencing and PICRUSt prediction, further confirmation is needed to improve the knowledge of the gut microbiota in *D. valens* and help to resolve taxonomic uncertainty at the species level.

## 1. Introduction

The red turpentine beetle, *Dendroctonus valens* LeConte (Coleoptera: Scolytidae), is a phloem-feeding insect that attacks pine trees. The beetle commonly attacks weakened or freshly dead trees, and it is often associated with more aggressive bark beetle species in its native region (North America and parts of Central America). In the early 1980s, *D. valens* was first found in *Pinus tabuliformis* forests in Northern China’s Shanxi province, where the pest’s first outbreak occurred in 1999 [1]. Since 1999, *D. valens* spread rapidly to the adjacent provinces of Shanxi and caused the death of more than 10 million healthy pine trees [2]. The widespread attacks and destructive damage of the beetle to pine forests in the past confirm that *D. valens* has become a primary pest in China.

Wood-eating insects harbor a large number of gut microbes and some of them can interact with a host to form a holobiont, which facilitates the beetles in overcoming a host tree’s nutritional limitation and chemical defenses [3,4,5,6,7]. For example, gut fungi in two longhorn beetles (*Anoplophora glabripennis* and *Saperda verstita*) were shown to help a beetle degrade the host’s wood [5,6]. Similar results were found for two other wood-ingesting beetles [8]; several typical nitrogen-fixing bacteria were isolated from *D. valens* gut, which may facilitate its growth and development by providing nitrogen [9]. Gut-associated bacteria of several beetle species (*D. valens*, *D. ponderosae*, and *Ips pini*) have been shown to be able to degrade host defensive monoterpenes in vitro [10,11,12]. In addition to mutualistic gut bacteria, bark beetles also harbor opportunistically pathogenic bacteria, for example, *Erwinia* bacteria can accelerate the mortality of *D. valens* once its larva has been infected by an entomopathogenic fungus, *Beauveria bassiana* [13]. The retention and persistence of these functional microorganisms in a beetle’s gut may directly relate to the fitness of the host insect. Bark beetles are a holometabolous insect whose gut condition will change along with its development [14,15]. However, whether and how these functional bacteria change during the development of *D. valens* remains unknown. Thus, a better characterization of a gut bacterial community will help to understand the role played by microbiota in the fitness of bark beetles and many other important aspects of its biology.

Historically, the gut bacteria of bark beetles were investigated using a traditional culture-based approach followed by clone-based Sanger sequencing of 16S rRNA [9,16,17,18,19,20,21,22]. During the last decade, next-generation sequencing technologies (e.g., Roche 454, Illumina MiSeq) have been widely used owing to the high throughput sequencing data and relatively low cost of these platforms, which have greatly benefitted our understanding of the diversity of the bacterial communities [23]. However, most of these studies (including the investigations of gut microbiota in bark beetles) sequenced short-read 16S rRNA gene amplicons (e.g., V1–V3, V4–V5) instead of the full-length gene sequences in gut bacterial community surveys, and the sequences can only be precisely identified by genus [24,25,26,27]. Although the complete sequencing of the 16S rRNA gene does not guarantee the identification of the species of a large proportion of the bacteria, a higher-resolution profile of gut bacterial communities is still needed to compromise the current taxonomic uncertainty of insects’ gut microbiota at the species level. 

Recently, a single-molecule real-time (SMRT) DNA sequencing system from Pacific Biosciences can generate raw reads with an average length of longer than 10 kb, and the method can also achieve comparable or even higher accuracy than other traditional platforms [28,29]. In this study, we utilized this technology to investigate gut bacterial communities throughout the ontogeny of *D. valens*, including young larvae, old larvae, dispersal adults, and colonized adults. 

## 2. Materials and Methods

### 2.1. Insects and Samples

Adult beetles were captured using Lindgren funnel traps that contained kairomone lure [(+)-α-pinene: (−)-β-pinene: (+)-3-carene = 1:1:1] (99%, 98%, 97%, respectively) (Sigma Aldrich, Shanghai, China) in the Tunlanchuan Forestry Station (N 37°48′, E 111°44′, average elevation 1400 m), Shanxi province. The rearing of *D. valens* followed the method we described in a previous study [30]. Uninfested *P. tabuliformis* trees (diameter ≥ 30 cm) were cut into 50 cm lengths, and both ends of the bolts were immediately dipped into melted paraffin to delay desiccation. Three evenly spaced holes (80 mm in diameter) were drilled into each bolt, and a pair of adult beetles was introduced into the holes separately. The holes were secured with wire mesh (mesh size, 2.0 mm), and checked every day until each pair of beetles entered the bark. The bolts were placed vertically in plastic boxes (50 cm diameter, 50 cm height). The containers were stored at room temperature/humidity throughout the rearing period [30]. 

After three weeks of rearing, the bark and phloem of one bolt were dissected and 10 young larvae were collected and placed in axenic plastic containers with moistened paper and then transported to the laboratory. Complete guts were stretched out by cutting the head and separating the abdomen, and only gut samples with a full gut track were used for further DNA extraction. After five weeks, 10 old larvae and their guts were collected using the method described above. The 10 adult beetles which emerged from the bolts were collected and referred to as newly emerged beetles. Another 10 beetles were randomly collected and then introduced to a healthy *P. tabuliformis* pine (the diameter at breast height is 40 cm). One week after the beetles entered the bark, the beetles and their guts were collected, referred to as colonized beetles [31].

### 2.2. DNA Extraction, PCR, PacBio Sequencing

Genomic DNA of gut tissues was extracted using the High Pure template PCR preparation kit (Roche Life Science, Indianapolis, IN, USA) according to the manufacturer’s instructions. Then, it was pulverized in liquid nitrogen using a Tissuelyser-24 (Jingxin Limited Company, Shanghai, China). The quality of DNA was assessed on 1% agarose gel, subsequently quantified using Quibit 3.0 (Thermo Fisher Scientific, Waltham, MA, USA), and diluted to 50 ng/μL for PCR amplification. Full-length 16S ribosomal RNA gene was amplified from 50 ng of genomic DNA using a bacteria-specific primer with symmetric barcodes. The same barcode (5′ to 3′) appears at both ends of an amplicon: 27 F (5-Barcode-AGAGTTTGATCMTGGCTCAG-3) and 1492 R (5′-Barcode-TACGGYTACCTTGTTACGACTT-3′). The PCR was performed using the following cyclical conditions: 94 °C for 2 min, followed by 30 cycles of 94 °C for 30 s, 55 °C for 30 s, and 72 °C for 90 s, and a final extension at 72 °C for 10 min. The amplicons were purified using the AMPure PB bead according to the recommended protocol, and quantities were assessed using an agilent bioanalyzer 2100. Barcoded amplicons were pooled for library construction, and SMRTbell library was prepared using PacBio DNA template preparation kit. The library was sequenced with PacBio RSII sequencer using C4 sequencing chemistry and P6 polymerase.

### 2.3. Sequence Data Analysis

PacBio raw reads were processed using a protocol called circular consensus sequencing (CCS) in SMRT analysis (2.3.0). Sequences shorter than 800 bp were removed before downstream analysis. The remaining effective reads were clustered into operational taxonomic units (OTUs) at 97% sequence similarity with a UPARSE algorithm [32]. Then, an NCBI BLAST tool was used to assign the 16S rRNA sequences to the taxonomical hierarchy at the species level, which was further confirmed manually. Rarefaction curves were estimated using QIIME pipeline. The number of reads was homogenized with respect to the sample with the lowest read counts, and alpha diversity indices, including phylogenetic diversity (PD, which represents the phylogenetic counterpart to species richness), Shannon diversity (represents species diversity), and Simpson diversity index (represents species evenness), were calculated for each sample using the ‘alpha_diversity.py’ script in QIIME. The representative sequences of all OTUs were used to construct neighbor-joining trees with QIIME. The phylogenetic tree, together with sample sequence abundance data, was used for weighted Unifrac principal coordinate analysis (PCoA), and a permutational multivariate analysis of variance based on the weighted UniFrac distance (PERMANOVA) was used to test for differences in community composition between two sample groups. Differences in relative abundance of Phyla and species between samples were analyzed using Kruskal–Wallis tests with multiple testing correction (false discovery rate, FDR) or following the Mann–Whitney *U* test using Bonferroni correction to adjust the probability.

The observed 16S rRNA gene sequences were clustered into OTUs using the ‘pick_closed_reference_outs.py’ script in QIIME, and the normalized OTU table was used to predict metagenomic functional content using the software package phylogenetic investigation of communities by reconstruction of unobserved states (PICRUSt) [33]. Kyoto Encyclopedia of Gene and Genomes (KEGG) databases were used to assign the functions of the predicted metagenome, and a graphic presentation was constructed using the R software ‘ggplot2’ package.

## 3. Results

### 3.1. Sequencing Information and Bacterial Diversity

We obtained a total of 109,303 reads from 32 *D. valens* gut samples, with an average of 3415 sequences per sample. These sequences were assigned to 289 bacterial OTUs. A total of 65 species were found in colonized adults, which is the highest amount among the four life stages, and these groups had the highest Shannon diversity index (Table 1). Shannon and Simpson diversity indices of gut microbiota in colonized adults were higher than the indices in beetles at other life stages, which suggested that colonized beetles had higher bacterial species richness and evenness of the gut bacterial communities than gut microbiota in beetles at other life stages. The Simpson diversity index of the bacterial community in dispersal adults is the lowest among the four life stages. No significant differences were found for observed OTUs and PD index between the gut bacterial communities in beetles at different life stages.

### 3.2. Bacterial Composition of Gut Samples at Different Life Stages

**Figure 1:**
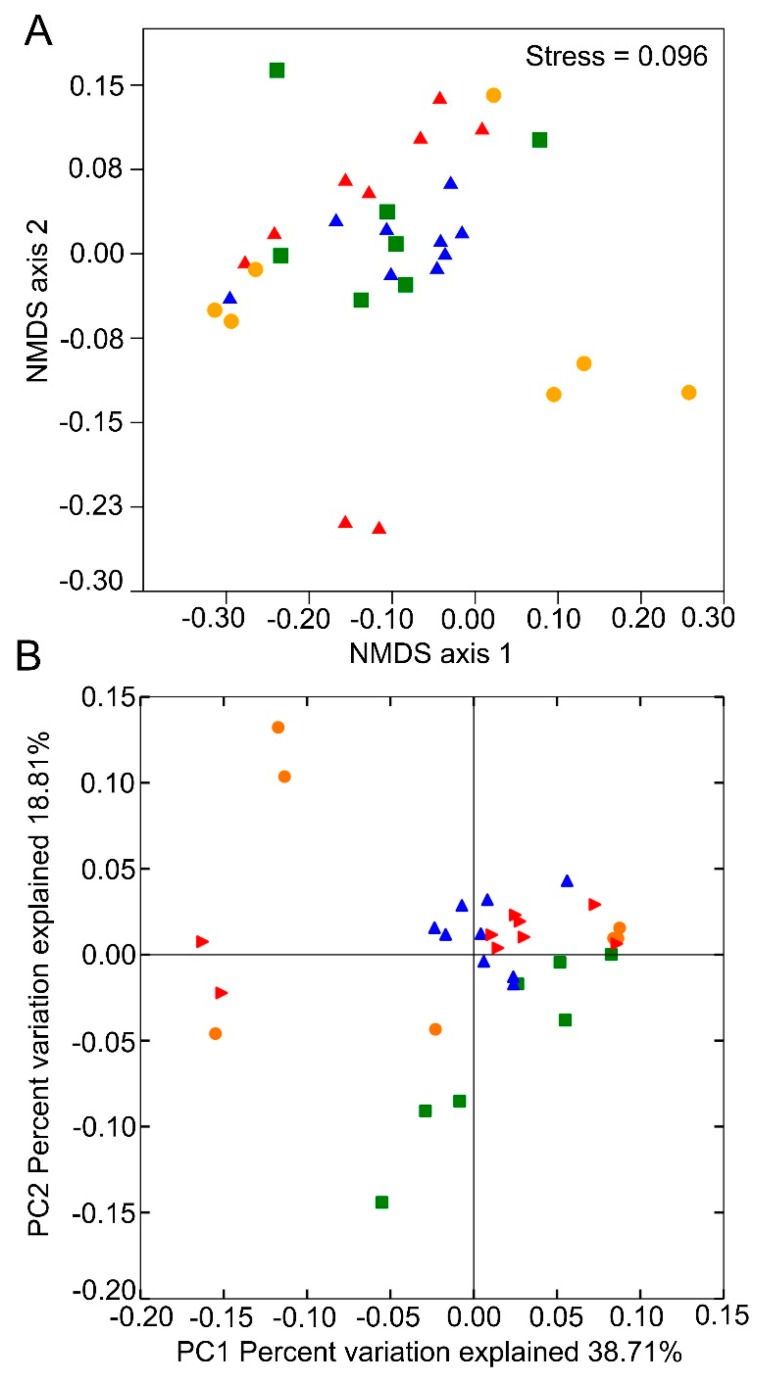
Non-metric multidimensional scaling (NMDS) and principal coordinates analysis (PCoA) of the bacterial communities in the gut of different life stages of *D. valens*. (**A**) Non-metric multidimensional scaling diagrams of 32 samples, based on Bray-Curtis distance matrix for bacterial communities that consisted of operational taxonomic units (OTUs) (97% similarity level). (**B**) Principal coordinate analysis plots based on the weighted UniFrac metric for bacterial communities. The red triangle represents young larval gut samples, the green square represents old larval gut samples, the orange circle represents dispersal adult gut samples, and the blue triangle represents female colonized adult samples.

In total, these OTUs were assigned to 8 bacterial phyla, 55 families, 102 genera, and 253 species. Based on the detected OTUs across the samples, a non-metric multidimensional scaling (NMDS) ordination analysis suggested that bacterial communities from *D. valens* gut at the four life stages are all clustered together, whereas gut samples between young larvae and colonized beetles clustered distinctively (Figure 1A, ANOSIM, R = 0.23, *p* = 0.016), and gut samples between old larvae and dispersal beetles clustered distinctively (Figure 1A, ANOSIM, R = 0.39, *p* = 0.003), which was confirmed by the NMDS diagram using the Bray–Curtis similarity metric. A phylogeny-based weighted UniFrac PCoA considering the relative abundance of OTUs showed no significant differences existing between groups, and was further corroborated by a dissimilarity test PERMANOVA (Figure 1B, PERMANOVA, *p* > 0.05).

**Figure 2:**
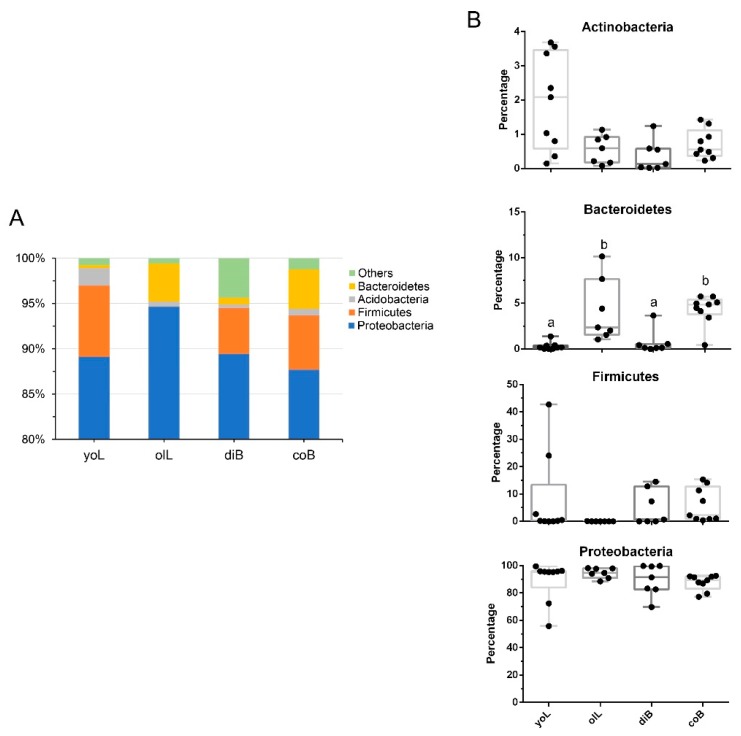
Phylum level composition. (**A**) Color-coded bar plot showing the average bacterial phylum distribution of the different groups sampled from gut communities of *D. valens* at different life stages (yoL, young larvae; olL, old larvae; diB, dispersal adults; coB, colonized adults). (**B**) The box shows the interquartile range of the relative abundance of the four main bacterial phyla from four groups: Proteobacteria, Firmicutes, Bacteroidetes, and Actinobacteria. Boxes represent the 25th and 75th percentiles. The solid line inside the box represents the median. Boxes with a different letter above are significantly different at p < 0.05.

At the phylum level, the most abundant bacterial phyla were Proteobacteria, Firmicutes, Acidobacteria, and Bacteroidetes, which are consistently predominant in *D. valens* gut at the four life stages (together representing more than 95% of the total sequences, Figure 2). The percentage of Bacteroidetes bacteria in guts of old larvae and colonized adults was significantly higher than that in young larvae guts and dispersed adult guts (Kruskal–Wallis test, X^2^ = 20.03, df = 3, *p* = 0.0002, Figure 2B). No significant differences were found for the other two predominant phyla among different groups.

**Figure 3:**
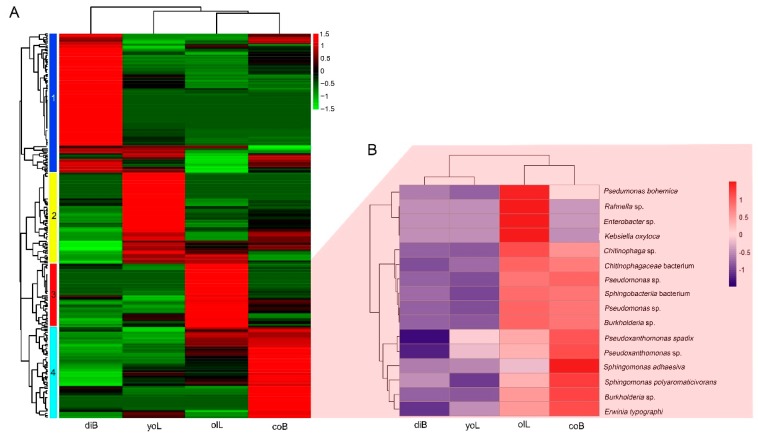
Species level composition. (**A**) Hierarchical clustering analysis of gut bacteria of *D. valens* at different life stages. Bacterial dendrogram is divided into four clusters that are marked by different color-coded boxes on the left (species in each cluster are listed in Table S1). (**B**) Heatmap showing 16 gut bacteria species with significant differences in abundance between groups (Kruskal–Wallis test, df = 3, FDR-corrected *p* < 0.05).

At the genus level, all 253 species from *D. valens* guts were subjected to two-dimensional hierarchical cluster analysis (Figure 3A, Table S1). Four major bacterial clusters were identified that exhibited a distinctly bacterial abundance pattern among different groups (Figure 3A, Table S1, Cluster 1, 91 species; Cluster 2, 60 species; Cluster 3, 60 species; Cluster 4, 42 species). The bacteria in Cluster 1 from dispersal adults’ guts had a higher abundance than those in the other three groups (Figure 3A, Table S1). Bacteria in Cluster 2, 3, 4 had a higher abundance in young larvae, old larvae, and colonized adults than those in the other three groups, respectively (Figure 3A, Table S1). Among these species, 17 gut bacterial species were found to have significant differences in abundance between the four groups (FDR-corrected Kruskal-Wallis test, df = 3, *p* values for each bacterium are listed in Table S1, Figure 3B).

### 3.3. Prediction of the Functional Metagenome of D. valens Gut Bacterial Communities

**Figure 4:**
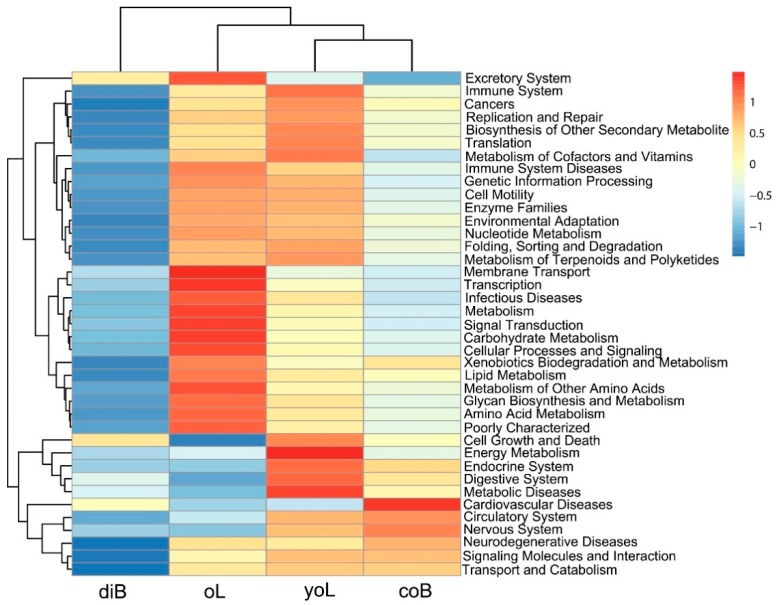
Heatmap of relative abundance of inferred functions by phylogenetic investigation of communities by reconstruction of unobserved states (PICRUSt) in the gut bacterial community of *D. valens* across its life cycle.

In order to better understand the metabolic function of *D. valens* gut microbiota, the functional metagenome was predicted using PICRUSt software (Figure 4). All of the predicted KEGG metabolic pathways were shown at the second hierarchical level. In all of the tested stages, bacterial communities seem to be rich in genes involved in amino acid metabolism, biosynthesis of other secondary metabolites, carbohydrate metabolism (Figure 4). In young larvae, predicted genes thought to be involved in energy metabolism, digestive system, and energy metabolism are more abundant than the other three life stages. In the old larvae, the relative abundance of several functional categories (e.g., carbohydrate metabolism, xenobiotics biodegradation and metabolism, amino acid metabolism) is increased compared to young larval stages. As for the gut bacterial communities in dispersal adults, most functional categories are decreased compared to the other three life stages (Figure 4).

## 4. Discussion

Bark beetles harbor a variety of gut bacteria and fungi, which display a wide diversity of interactions with insect hosts, ranging from obligate mutualism to pathogen. Thus, accumulating studies have described the microbial diversity in bark beetle’s gut. However, most of these studies are based on a culture-dependent method or next-generation sequencing technologies (e.g., Roche 454, Illumina MiSeq). The former method cannot isolate and culture all bacterial species that are alive in the insect’s gut, and the next-generation sequencing platform generates relatively short sequences, mostly 400–500 bp, which cannot be assigned to bacterial species. To date, this study is the first to utilize the high-throughput, long-read sequencing capability of the PacBio system to obtain full-length 16S sequences for gut microbial diversity in bark beetles. We obtained a total of 253 bacterial species by using the platform, which is obviously more abundant than the studies that utilized culture-dependent methods [9,16,20]. Furthermore, this investigation will expand the knowledge of how the gut bacterial community of bark beetles and its potential function change at different developmental stages. However, there is still 1.25%–5.52% of sequences that cannot be assigned to a genus or species (Table S1), which suggests that even the complete sequencing of the 16S rRNA gene does not guarantee the identification of species of all bacteria in *D. valens* gut, although the taxonomic uncertainty in the study is obviously less than that of previous studies basing on Illumina and 454 sequence data [30,34].

Bark beetles belong to holometabolous insects, and their guts are unstable habitats due to the several times molting during larval development, which sheds foregut, hindgut, and attached bacterial populations [14,15]. Besides, the metamorphosis process would further eliminate the entire larval gut and contents [15]. Thus, bacterial community composition at different life stages should be varied logistically. For example, several bacterial species, including *Lactococcus lactis*, *Acinetobacter* sp., and *Pantoea vagans*, were not detected in one or two life stages of *D. valens*, whereas they are readily isolated from the beetle’s frass or galleries [16,35]. Thus, the newly molted insects may acquire new bacterial species from the environment during food ingestion. Alpha diversity results showed that gut bacterial communities in colonized beetles have a greater species richness than those in other life stages, whereas greater values of Simpson diversity index also indicate a greater dominance of the community by certain species, that is, lower evenness, which is partly caused by a larger number of bacterial species in colonized beetles (~180 species) than dispersal adults (~160 species) (Table 1, Table S1). These extra 20 bacterial species, including *Acinetobacter* sp., *Comamonas testosterone*, *Asaia platycodi*, and *Gluconobacter cerinus*, may acquire from food when beetles colonize pine trees when compared to dispersal beetles. Here, we did not compare bacterial compositions between the two sexes of *D. valens*, mainly because our previous results have shown that male and female have similar gut microbiota compositions using both culture-dependent and high-throughput pyrosequencing methods [16,34,36].

Previous studies have shown that the β-diversity changes during the beetle’s life cycle [19,26,37]. In this study, NMDS showed that bacterial community structure in colonized beetles is different to those in young larvae. However, PCoA showed that the bacterial community structure from young larvae, old larvae, dispersal adults, and colonized adults have no significant differences (Figure 1), suggesting that *D. valens* harbors a relatively stable gut bacterial community. This may partly be caused by its life history strategy. *D. valens* lives and feeds in pine-tree phloem for most of their life cycles (eggs, pupae, larvae, newly emerged adults, and colonized adults) [14], which enables the insects to directly transfer gut microbes between conspecifics. The different platforms used in the current study and previous studies may also cause the variation. Furthermore, previous studies found that biases of bacterial communities can be readily introduced by different primer choices or DNA extraction methods [38,39,40,41,42], though one study showed that DNA extraction protocols cause differences in 16S rRNA amplicon sequencing efficiency instead of bacterial community profile composition [43]. Fortunately, we used a standardized approach to compare the bacterial populations in *D. valens* guts between different life stages with a pair of primers. However, the variation of the achieved results between this study and previous studies using different primers or different methods need further confirmation, for example, using PCR-independent data to assess biases.

Our PacBio SMRT sequencing results showed that Proteobacteria is a dominant phylum, though a total of 253 species were found, and bacteria in Bacteroidetes, Firmicutes, and Actinobacteria phylum have relatively lower abundance (Figure 2). A similar result was found for the gut bacterial communities of the other 11 *Dendroctonus* species, which were investigated using 454 pyrosequencing analysis [25]. At the species level, *Pseudomonas* sp., *Klebsiella oxytoca*, *Serratia liquefaciens*, *Burkhodreia* sp., and *Frateuria aurantia* are dominant taxa (Figure 3, Table S1), which persistently existed in *D. valens* gut throughout its life cycle (Figure 3). The results concur with previous studies, as these dominant taxa, except for *F. aurantia*, are widely found in bark beetles’ gut, and the results utilized either culture-dependent or culture-independent methods to investigate bacterial community. Similarly, several core bacteria were found to persist at all life stages for other bark beetles including *D. rhizophagus*, *D. armandi*, and *I. pini*, though some bacterial species are less common at a certain life stage [21,26,37]. *F. aurantia* is a species of pseudo-acetic acid bacteria [44], which is often isolated from flowers and was first reported in the bark beetle’s gut. The bacteria have a higher abundance in young larvae gut than those in *D. valens* gut at the other three life stages (Table S1). In the future, we may try to isolate the bacteria and explore its biological function and the reason why its abundance changed between different life stages.

Our previous studies and other studies have used culture-dependent methods to isolate bacterial species in the gut of several *Dendroctonus* beetles and to further evaluate the functional role of certain bacteria, for example, pheromone production, nitrogen fixation, digestive processes, plant defensive compound detoxification, and interaction with other associated fungi [3,11,12,13,16,45]. However, more bacterial functions remain unknown, as only parts of gut bacteria were isolated and identified from bark beetles by comparing the results between culture-dependent methods and next-generation sequencing platforms. Considering the time-consuming nature of culture-based methods, PICRUSt may provide some clues for predicting the metabolic capacities of the gut bacterial community. In both larval stages and colonized beetles, predicted genes of bacterial communities are rich in genes involved in the digestive system, energy metabolism, transport and catabolism, and carbohydrate metabolism (Figure 4). A similar pattern was found for *D. rhizophagus*. The nitrogen fixation and cellulose breakdown bacteria are closely associated with the beetle [19]. Even under attack by bark beetles, the contents of carbon and nitrogen (D-fructose and D-glucose) in pine trees showed no decrease than those in healthy trees [30,45]. Meanwhile, the most functional categories of the bacterial community in dispersal adults decreased compared to the other three life stages (Figure 4). We hypothesize that the dispersal beetles have fewer food supplies than colonized adults, which leads to less-abundant microbiota in beetles’ guts [46]. Furthermore, the concentration of defensive hydrocarbons (e.g., monoterpenes) rose rapidly at the onset of a beetle attack and reached a maximum in their gallery surroundings [30,47,48], which is toxic to both beetles and their associated microbes [10,49,50,51]. The microbiome in larvae was more enriched for predicted genes thought to be involved in the metabolism of terpenoids and polyketides, and xenobiotics biodegradation, which may be explained by their intensive feeding on pine phloem that contains harmful chemicals. However, we must be very cautious about these conclusions and admit that deducting the biofunctions of gut bacteria from metagenomic DNA has a serious limitation, as the method does not permit the recognition of functionally active species. Thus, studies that complement massive sequencing with RNA sequencing and bacterial cultivable fractions are important at this point.

## 5. Conclusions

This study used a PacBio sequencing system to characterize *D. valens* gut microbiota throughout its ontogeny, and identified a total of eight phyla, 55 families, 102 genera, and 253 species. Colonized beetles have the richest bacterial community and the lowest evenness in all life stages. *Pseudomonas* sp., *S. liquefaciens* may serve as members of the core bacteriome. Gut bacteria in both larvae and colonized beetles are rich in xenobiotic and terpenoid biodegradation, which are decreased in dispersal beetles. The study improves the knowledge of the gut microbiota in *D. valens* at the species level.

## Figures and Tables

**Table 1:** Richness and diversity estimation (mean ± SEM) of the bacterial community in the gut at different life stages of *Dendroctonus valens*.

Sample	Species Richness Indices		Species Diversity Indices	
	OTUs Observed	Phylogenetic Diversity	Shannon Diversity	Simpson Diversity
Young larvae (n = 9)	45.89 ± 3.08	3.01 ± 0.21	2.40 ± 0.21	0.63 ± 0.06
Old larvae (n = 7)	53.14 ± 3.36	2.82 ± 0.15	2.71 ± 0.22	0.66 ± 0.05
Dispersal adults (n = 7)	45.29 ± 7.93	2.74 ± 0.40	2.56 ± 0.65	0.58 ± 0.14
Colonized adults (n = 9)	65.33 ± 4.30	3.58 ± 0.21	3.24 ± 0.29	0.73 ± 0.05

## Supplementary Materials

The following are available online at https://www.mdpi.com/2075-4450/10/8/248/s1, Table S1: Relative abundances of all identified species in *D. valens* at different life stages.
